# Bioactive Glass-Based Materials Versus Conventional Materials for Primary Tooth Restorations: A Comparative Meta-Analysis

**DOI:** 10.7759/cureus.113767

**Published:** 2026-08-01

**Authors:** Abdulaziz Zailai, Abdullah Noor, Dalal Alkhawari, Selwan Elsherbeny, Lubana Alsaffar, Budour O Alshaghdali, Rahaf Alsakran, Yousef A Al-Sultan, Nasser F Aldhumayri, Walaa Alqudaihi, Aisha A Adawi, Tuqa I Alobayshi, Shahad A Althamin, Ali S Metwaly

**Affiliations:** 1 Restorative Dentistry, Jazan Specialized Dental Center, Jazan, SAU; 2 Pedodontics, King Abdulaziz University, Jeddah, SAU; 3 Dentistry, Ministry of Health - Kuwait, Kuwait City, KWT; 4 Pedodontics and Oral Health, Faculty of Dental Medicine for Girls, Al-Azhar University, Cairo, EGY; 5 General Dentistry, College of Dentistry, AlFaraby Medical Center, Dammam, SAU; 6 Dentistry, Primary Health Care Center, Al Humran, Al-Baha Health Cluster, Al-Baha, SAU; 7 General Dentistry, Majmaah University, Riyadh, SAU; 8 Dental Public Health, Ministry of Health - Saudi Arabia, Riyadh, SAU; 9 General Dentistry, Safwat Amina Medical Company, Sakaka, SAU; 10 Dentistry, Riyadh Elm University, Riyadh, SAU; 11 Dentistry, Jazan University, Jazan, SAU; 12 Dentistry, Al-Nabhaniyah General Hospital, Al-Nabhaniyah, SAU; 13 Pediatric Dentistry, Ministry of Health - Saudi Arabia, Riyadh, SAU; 14 Precision Medicine, Faculty of Pharmacy, Alexandria University, Alexandria, EGY

**Keywords:** bioactive materials, biomimetics, composite resins, dental caries, glass ionomer cements, meta-analysis, pediatric dentistry, primary teeth

## Abstract

The clinical management of carious primary teeth is complicated by distinct morphological features and behavioral challenges. Bioactive glass-based materials are biomimetic alternatives to conventional restorations, with promising remineralization potential and improved mechanical durability. This systematic review and meta-analysis aimed to evaluate the clinical efficacy of bioactive materials compared with that of conventional restorative materials in primary dentition. A protocol-driven (International Prospective Register of Systematic Reviews (PROSPERO): CRD420261329678), Preferred Reporting Items for Systematic Reviews and Meta-Analyses 2020 (PRISMA 2020)-compliant search was conducted across six major databases and grey literature up to May 2026. Randomized controlled trials (RCTs) comparing bioactive materials (e.g., ACTIVA BioACTIVE, Cention N, glass hybrids) with conventional materials (e.g., resin-modified glass ionomer cements, composites, compomers) with a minimum six-month follow-up were included. The risk of bias was assessed using RoB 2 (The Cochrane Collaboration, London, UK). A random-effects meta-analysis utilizing the restricted maximum likelihood estimator with Hartung-Knapp-Sidik-Jonkman adjustment was performed for clinical success. The certainty of evidence was appraised using the Grading of Recommendations Assessment, Development, and Evaluation (GRADE) framework. Seventeen studies (16 RCTs and 1 in vitro) were included. For the primary outcome, 16 RCTs comprising 1,293 randomized pediatric participants and 1,028 successful events were pooled. No statistically significant difference in overall clinical success/retention was observed between bioactive and conventional materials (RR = 1.00; 95% CI: 0.96 to 1.04; p = 0.9635; I² = 50.8%). Subgroup analyses by comparator class and meta-regression for follow-up duration revealed no significant effect modification. Secondary analyses indicated that bioactive materials may significantly reduce operator placement time (mean difference = -2.37 minutes; p < 0.0001). The overall certainty of the evidence for clinical success was rated as high. Bioactive materials demonstrate clinical efficacy and retention rates equivalent to those of established conventional restorative materials in primary teeth. Given their non-inferior clinical performance and potential workflow efficiencies, bioactive materials are a highly viable and biomimetic option for pediatric restorative dentistry.

## Introduction and background

Dental caries is one of the most prevalent chronic diseases affecting children globally and requires efficacious and durable restorative interventions [1]. The management of carious primary teeth requires highly specific clinical considerations due to their distinct anatomical and histological characteristics, including thinner enamel and dentin, disproportionately larger pulp chambers, and a shorter functional lifespan than permanent dentition [1].

Amalgam was the gold standard for posterior restorations; however, rising aesthetic demands and sustained environmental concerns have catalyzed a shift toward tooth-colored alternatives [2,3]. Conventional materials, such as glass ionomer cements (GICs) and composite resins, are the mainstays of pediatric restorative dentistry. GICs are highly advantageous because of their biocompatibility, chemical adhesion to dental hard tissues, and cariostatic effects mediated by long-term fluoride release [4,5]. However, their application, particularly in load-bearing Class II cavities, is constrained by suboptimal mechanical properties, including low fracture toughness, brittleness, and poor wear resistance [6,7]. While conventional composite resins exhibit superior aesthetics and mechanical strength, they are technique-sensitive, require moisture control, undergo polymerization shrinkage, and lack intrinsic remineralization capabilities [8].

To bridge the functional gap between conventional GICs and composite resins, hybrid formulations such as resin-modified glass ionomer cements (RMGICs), nanofilled RMGICs, and compomers (polyacid-modified resin composites) have been developed [9,10]. Long-term clinical evaluations have demonstrated that RMGICs and compomers provide improved handling characteristics, enhanced marginal integrity, and higher overall clinical success rates than conventional GICs [10-12]. Challenges such as secondary caries incidence, restoration retention failure, and microleakage remain persistent issues in the restorative management of primary teeth [7,8].

Recently, the shift toward biomimetic and regenerative dentistry has introduced bioactive glass-based materials, such as bioactive composites (e.g., ACTIVA), bioactive glass ionomers, and biosilicate cements, as therapeutically superior alternatives [1]. Unlike traditional restorative materials, which primarily function as passive fillers, bioactive materials interact with the patient's oral environment. They are characterized by their capacity for the continuous release and recharge of calcium, phosphate, and fluoride ions, which promotes the remineralization of demineralized dentin and enamel, enhances cellular biocompatibility, and provides superior resistance against microleakage at the restorative margins [1].

Despite the theoretical and in vitro advantages of bioactive materials, existing clinical evidence regarding their comparative effectiveness against conventional materials remains fragmented across various individual randomized controlled trials (RCTs) and observational studies. There is a critical need to synthesize these data to formulate evidence-based guidelines for pediatric dental practitioners. Therefore, this systematic review and meta-analysis aimed to evaluate and compare the clinical efficacy of bioactive materials vs. conventional materials in the management of carious primary teeth, specifically focusing on overall clinical success rates, restoration retention, marginal integrity, and the incidence of secondary caries. Addressing this fragmented data is of high clinical significance; synthesizing these outcomes will help close current knowledge gaps and provide pediatric dental practitioners with evidence-based guidelines for adopting these biomimetic alternatives.

## Review

Methods

Protocol Registration and Reporting Format

This systematic review and meta-analysis was conducted in adherence to the updated Preferred Reporting Items for Systematic Reviews and Meta-Analyses 2020 (PRISMA 2020) guidelines [13]. The a priori protocol was prospectively registered in the International Prospective Register of Systematic Reviews (PROSPERO) database under registration number CRD420261329678 [14].

Eligibility Criteria

The eligibility criteria were established using the PICOS (population, intervention, comparator, outcomes, and study design) framework. The population was children aged 3-12 years presenting with cavitated carious lesions in primary teeth (deciduous dentition) requiring direct restorative treatment. The intervention comprised bioactive glass-based restorative materials, including bioactive composite resins (e.g., ACTIVA BioACTIVE), bioactive glass ionomers, giomers, and biosilicate cements exhibiting documented ion release or remineralization capacity. The comparator comprised conventional materials, including conventional composite resins (incremental or bulk-fill), amalgam, RMGICs, high-viscosity glass ionomer cements (HVGICs), and compomers.

The primary outcomes were overall clinical restoration success and retention rates over a minimum six-month follow-up period; secondary outcomes included marginal integrity, incidence of secondary caries, microleakage, and operator placement time. RCTs (parallel-group or split-mouth designs) and prospective controlled clinical trials published between January 2010 and May 2026 were included, while retrospective studies, case reports, and studies lacking standard clinical evaluation criteria (e.g., USPHS, Fédération Dentaire Internationale (FDI), Ryge) were excluded.

Information Sources and Search Strategy

A multimodal search strategy was executed across six major electronic databases: PubMed/MEDLINE, Embase, Cochrane Central Register of Controlled Trials (CENTRAL), Scopus, and the Web of Science. The search syntax integrated Medical Subject Headings (MeSH), Emtree terms, and free-text keywords optimized with Boolean operators (AND, OR, NOT) and truncation wildcards. The keywords included combinations of "bioactive glass", "Activa Bioactive", "primary teeth", "deciduous dentition", "restorative materials", and "clinical efficacy". To mitigate publication bias, grey literature was searched via ClinicalTrials.gov and the WHO International Clinical Trials Registry Platform (WHO-ICTRP). Furthermore, forward and backward citation tracking (snowballing) of the included studies was conducted. There were no language restrictions.

Study Selection and Data Extraction

Following deduplication, two independent reviewers screened the titles and abstracts of the records retrieved. Full-text articles of potentially eligible studies were evaluated against predefined inclusion criteria. Discrepancies were resolved through arbitration by a third reviewer. The selection process was mapped using a PRISMA 2020 flow diagram. The inter-rater reliability for study inclusion was quantified using Cohen’s kappa coefficient (κ) [15]. Data extraction was performed independently using a standardized, pilot-tested form that captured study characteristics, demographics, cavity classifications (Class I vs. Class II), material specifications, follow-up duration, and primary/secondary outcome metrics.

Quality Assessment and Risk of Bias

The methodological quality and risk of bias of the included studies were independently appraised by two reviewers. For RCTs, RoB 2 (The Cochrane Collaboration, London, UK) was utilized to evaluate the randomization process, deviations from intended interventions, missing outcome data, measurement of the outcome, and selection of the reported results [16]. For non-randomized comparative studies, the Risk of Bias in Non-randomized Studies of Interventions (ROBINS-I) tool was applied [17].

Statistical Analysis and Data Synthesis

All statistical analyses were performed using the R statistical software environment version 4.6.0 (R Foundation for Statistical Computing, Vienna, Austria) [18], utilizing the meta [19] and metafor [20] packages.

For dichotomous outcomes (e.g., clinical success and retention failure), pooled effect sizes were calculated as risk ratios (RR) with 95% confidence intervals (CIs). For continuous outcomes (e.g., operator placement time), mean differences (MD) or standardized mean differences (SMD) with 95% CIs were computed.

Given the anticipated clinical and methodological variability among the different biomaterials and cavity preparations, a random-effects (RE) model was employed. The between-study variance (τ²) was estimated using the restricted maximum likelihood (REML) method [21]. To account for uncertainty in estimating between-study heterogeneity and minimize the false-positive rate inherent in meta-analyses with a limited number of studies, the Hartung-Knapp-Sidik-Jonkman (HKSJ) variance adjustment was systematically applied [22].

Heterogeneity Assessment and Exploration

Statistical heterogeneity was quantified using Cochran’s Q test (p < 0.10 indicating statistical significance), the I² statistic (where >50% signifies substantial heterogeneity), and τ² [23]. To explore the potential sources of heterogeneity, predefined subgroup analyses were conducted based on cavity classification (Class I vs. Class II) and comparator material type (e.g., RMGIC vs. conventional composites). Additionally, a leave-one-out sensitivity analysis was performed to test the robustness of the pooled estimates and to identify any single study exerting an undue influence on the overall effect size.

Publication Bias and Certainty of Evidence

Where ≥10 studies were available for a specific outcome, reporting bias and small-study effects were visually assessed using contour-enhanced funnel plots [24] and formally quantified using Egger’s regression test [25]. The overall certainty of the synthesized evidence was evaluated using the Grading of Recommendations Assessment, Development, and Evaluation (GRADE) framework [26]. Evidence was downgraded based on limitations in study design, inconsistency, indirectness, imprecision, or publication bias, culminating in a summary of findings table to facilitate clinical decision-making.

Results

Study Selection

**Figure 1:**
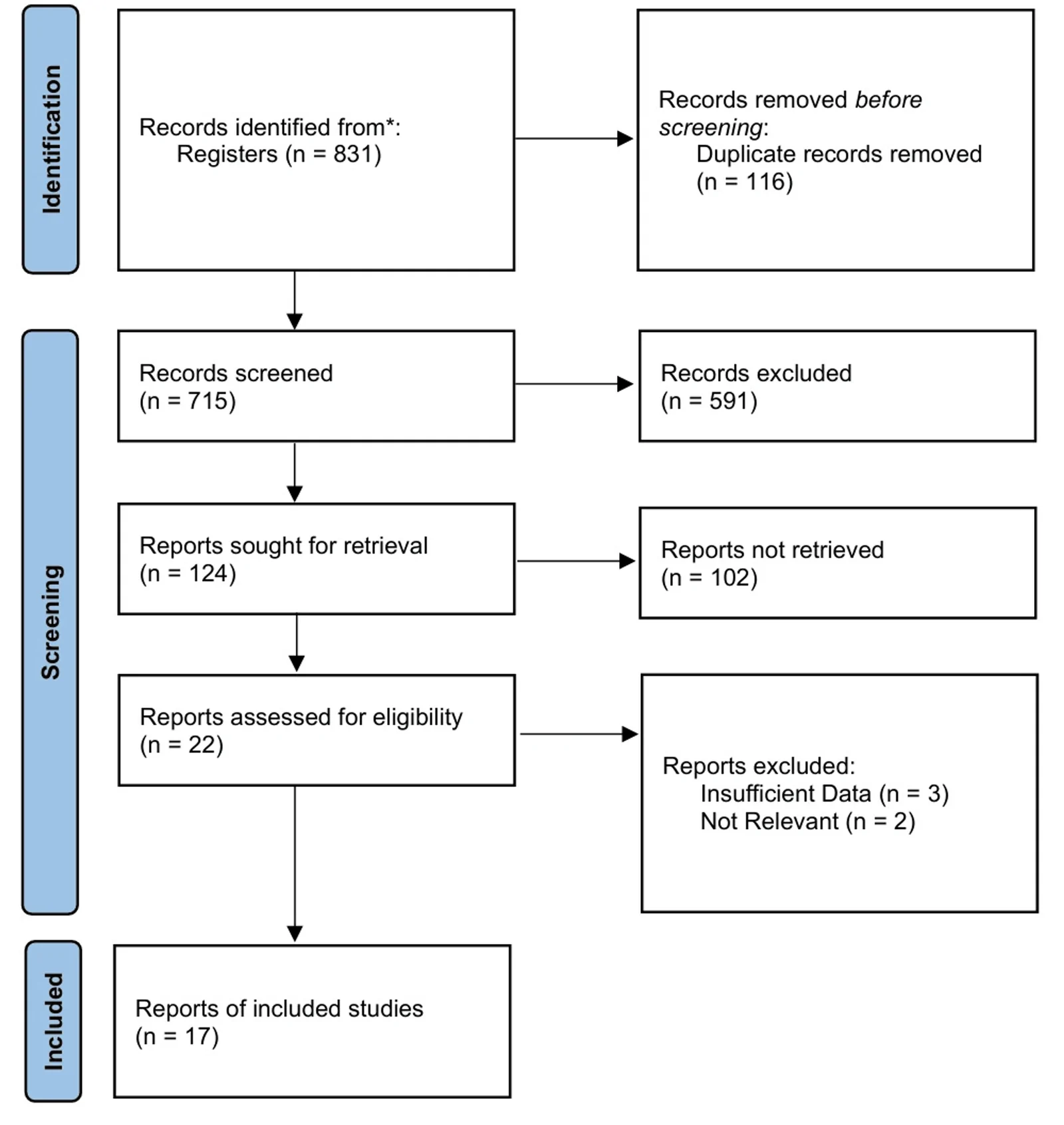
PRISMA 2020 flow diagram detailing the study selection process PRISMA: Preferred Reporting Items for Systematic Reviews and Meta-Analyses

The initial systematic search of the designated electronic databases and trial registries yielded 831 records. After removing 116 duplicate entries, the titles and abstracts of 715 unique records were screened, leading to the exclusion of 591 records. Subsequently, 124 full-text reports were sought, of which 102 could not be retrieved or were not available in full text. The remaining 22 full-text reports were assessed using the predefined eligibility criteria. Five studies were excluded (three due to insufficient/unextractable data and two due to a lack of clinical relevance to the specific PICO criteria, such as evaluating adult populations). Seventeen studies (16 RCTs and one in vitro study) met the inclusion criteria and were included in the systematic review and meta-analysis. The study selection process, including the reasons for exclusion, is detailed in the PRISMA 2020 flow diagram (Figure 1). The inter-rater reliability for the full-text screening phase was substantial, with a κ of 0.754 (95% CI: 0.49-1.02; observed agreement: 90.0%).

Study Characteristics

The 16 included clinical trials enrolled and randomized 1,293 pediatric participants. The studies evaluated various bioactive glass-based interventions, including ACTIVA BioACTIVE, Equia Forte, and Giomer-based composites. The comparator groups comprised conventional materials, primarily RMGICs, HVGICs, compomer, and conventional resin composites. The follow-up duration ranged from three to 36 months (Table 1).

**Table 1:** Characteristics of included studies RCT: randomized controlled trial, ART: atraumatic restorative treatment, HVGIC: high-viscosity glass ionomer cement, RMGIC: resin-modified glass ionomer cement, LC-GIC: light-cured glass ionomer cement, USPHS: United States Public Health Service criteria, FDI: Fédération Dentaire Internationale criteria, SBS: shear bond strength

Study (author, year)	Study design	Population and sample size (age range)	Intervention (bioactive/test material)	Comparator (conventional/control material)	Follow-up duration	Primary outcomes assessed
Pássaro et al., 2022 [27]	Parallel RCT	182 children; 182 primary molars (4-8 y)	Giomer (Beautifil Bulk Restorative)	HVGIC (Equia Forte)	24 months	Restoration survival (FDI/Roeleveld criteria)
Bhavana et al., 2024 [28]	Split-mouth RCT	31 children; 62 primary molars (5-8 y)	Bioactive Composite (ACTIVA BioACTIVE)	RMGIC	12 months	Marginal discoloration, surface texture, retention (USPHS)
Olegário et al., 2019 [29]	Parallel RCT	568 children; 568 primary molars (4-7 y)	Glass Carbomer	Compomer (Dyract) and HVGIC (Equia Fil)	36 months	Restoration survival of ART (ART criteria)
Lardani et al., 2022 [30]	Split-mouth RCT	45 children; 179 primary molars (5-9 y)	Bioactive Composite (ACTIVA BioACTIVE)	Bulk-fill Composite (SDR)	12 months	Esthetic, functional, biological properties (FDI)
Deepika et al., 2022 [31]	Split-mouth RCT	50 children; 100 primary molars (5-9 y)	Bioactive Composite (ACTIVA BioACTIVE)	Giomer (Beautifil Flow Plus)	12 months	Marginal integrity, retention, color match (USPHS)
da Cunha et al., 2022 [32]	Split-mouth RCT	27 children; 107 primary molars (4-9 y)	Alkasite (Cention N) w/ and w/o adhesive	RMGIC (Vitremer)	12 months	Restoration survival rate
Malik et al., 2025 [33]	Parallel RCT	16 children; 32 primary incisors (3-6 y)	Glass Hybrid (GC Equia Forte HT)	Composite (GC G-aenial)	3 months	Clinical effectiveness (FDI), parental satisfaction
Banon et al., 2024 [34]	Split-mouth RCT	21 children; 86 primary molars (5-10 y)	Bioactive Composite (ACTIVA BioACTIVE)	Compomer (Dyract eXtra)	24 months	Clinical and radiographic success (USPHS), operator placement time
Shihabi et al., 2026 [35]	Split-mouth RCT	20 children; 40 primary molars (6-9 y)	Bioactive Composite (ACTIVA BioACTIVE)	Composite (Tetric N-Ceram)	12 months	Clinical success, marginal adaptation, retention (USPHS)
Akman and Tosun, 2020 [36]	Parallel RCT	30 children; 160 primary molars (6-10 y)	Glass Hybrid (Equia Fil)	Bulk-fill composites (SonicFill, X-tra fil) and Nano-hybrid (Filtek)	12 months	Retention, marginal adaptation, secondary caries (USPHS)
Shagale et al., 2020 [37]	Split-mouth RCT	39 children; 78 primary molars (5-9 y)	Glass Hybrid (Equia Forte)	Bulk-fill composite (Tetric N-Ceram)	12 months	Esthetic, functional, biological properties (FDI)
Kataria et al., 2023 [38]	Parallel RCT	43 children; 75 primary molars (3-8 y)	Glass Hybrid (GC 9 Extra) and Alkasite (Cention N)	HVGIC (Ketac Universal)	12 months	Clinical and radiographic success (USPHS)
Beltagy and Elhatery, 2018 [39]	Split-mouth RCT and In vitro	15 children; 30 primary molars (4-7 y)	Bioactive Composite (ACTIVA BioACTIVE)	RMGIC (Vitremer)	12 months (Clinical)	Clinical success (USPHS), SBS
Mansour et al., 2024 [40]	Split-mouth RCT	24 children; 48 primary molars (4-6 y)	Glass Hybrid (Equia Forte)	RMGIC (Riva LC)	12 months	Marginal discoloration, integrity, wear, sensitivity (USPHS)
Inthihas et al., 2019 [41]	Parallel RCT	51 children; 102 primary molars (4-8 y)	Giomer (Beautifil II) and Nano-ionomer (Ketac Nano)	LC-GIC (Fuji II LC)	12 months	Clinical and radiographic success (FDI)
Sengul and Gurbuz, 2015 [42]	Parallel RCT	41 children; 146 primary molars (5-7 y)	Giomer (Beautifil)	Compomer, RMGIC, and Hybrid Composite	24 months	Restoration failure rate (FDI)
Bhatia et al., 2022 [43]	In vitro	12 extracted primary molars	Bioactive Composite (ACTIVA BioACTIVE)	RMGIC (Fuji II LC)	21 Days (in vitro)	SBS, calcium ion release

Risk of Bias Assessment

**Figure 2:**
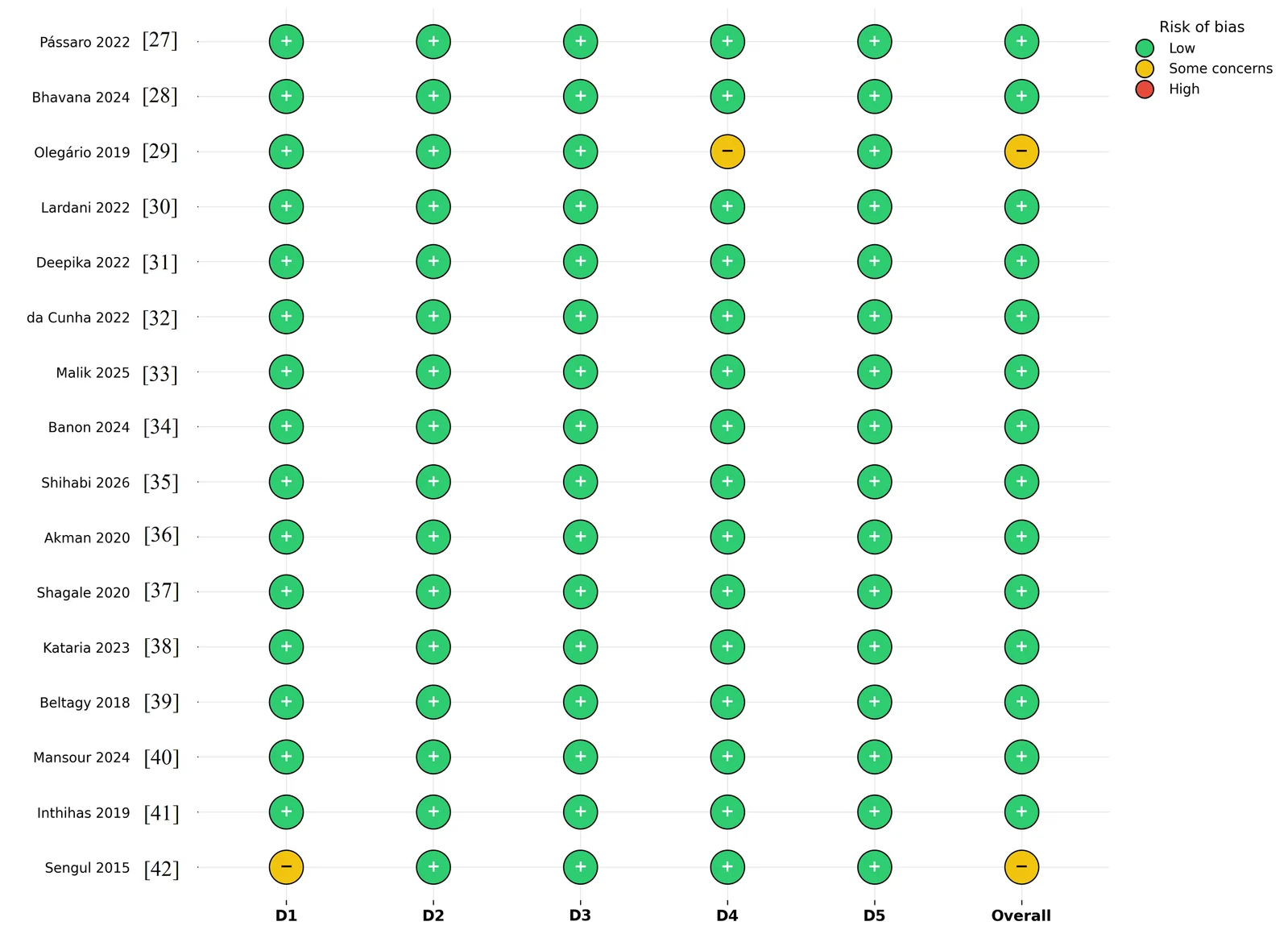
RoB 2 The traffic-light plot illustrates domain-level and overall bias judgments for the 16 included clinical trials [27-42].

**Figure 3:**
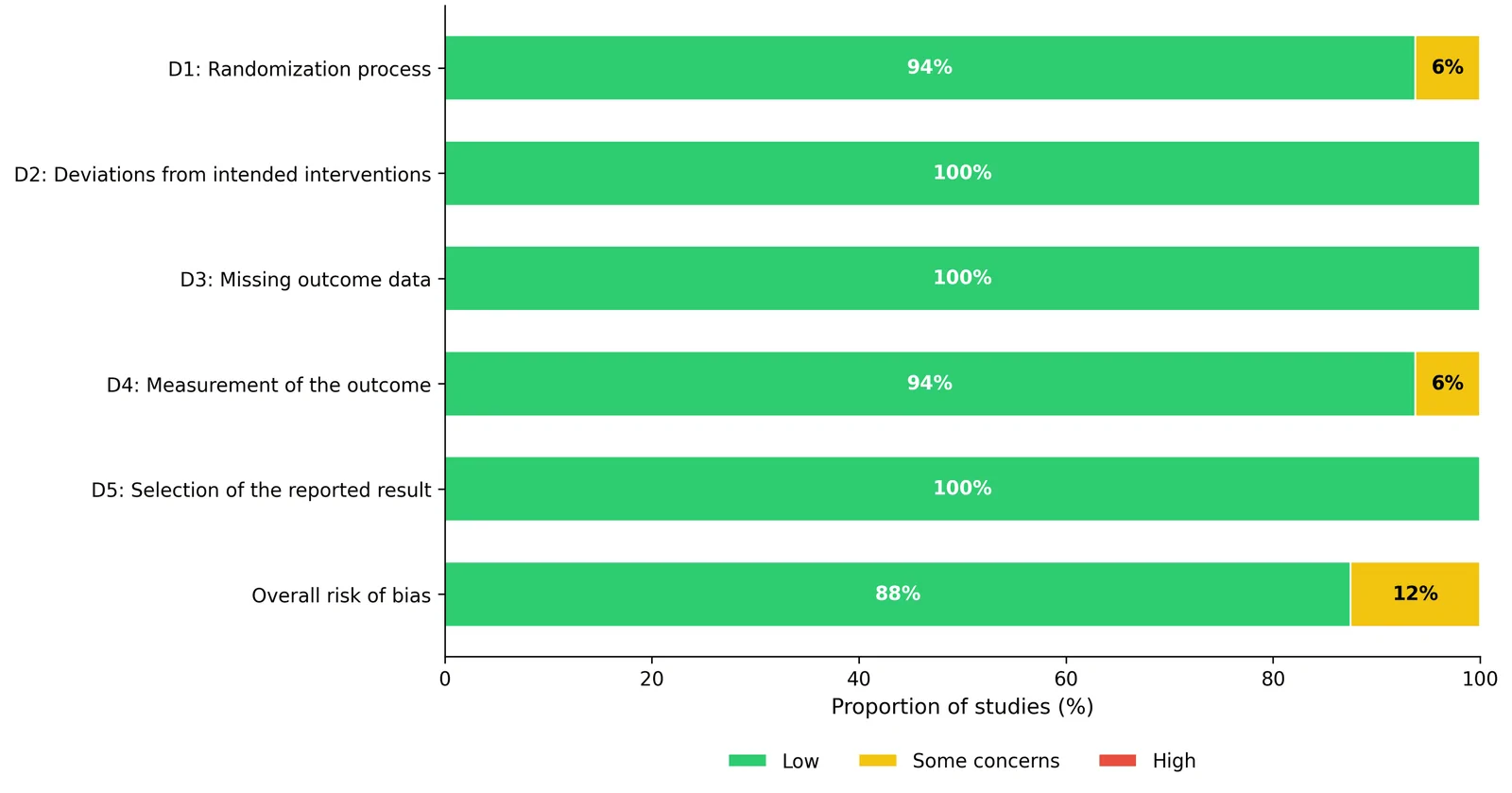
RoB 2 summary The bar plot shows the proportion of studies with low risk, some concerns, and high risk of bias across the five domains.

**Figure 4:**
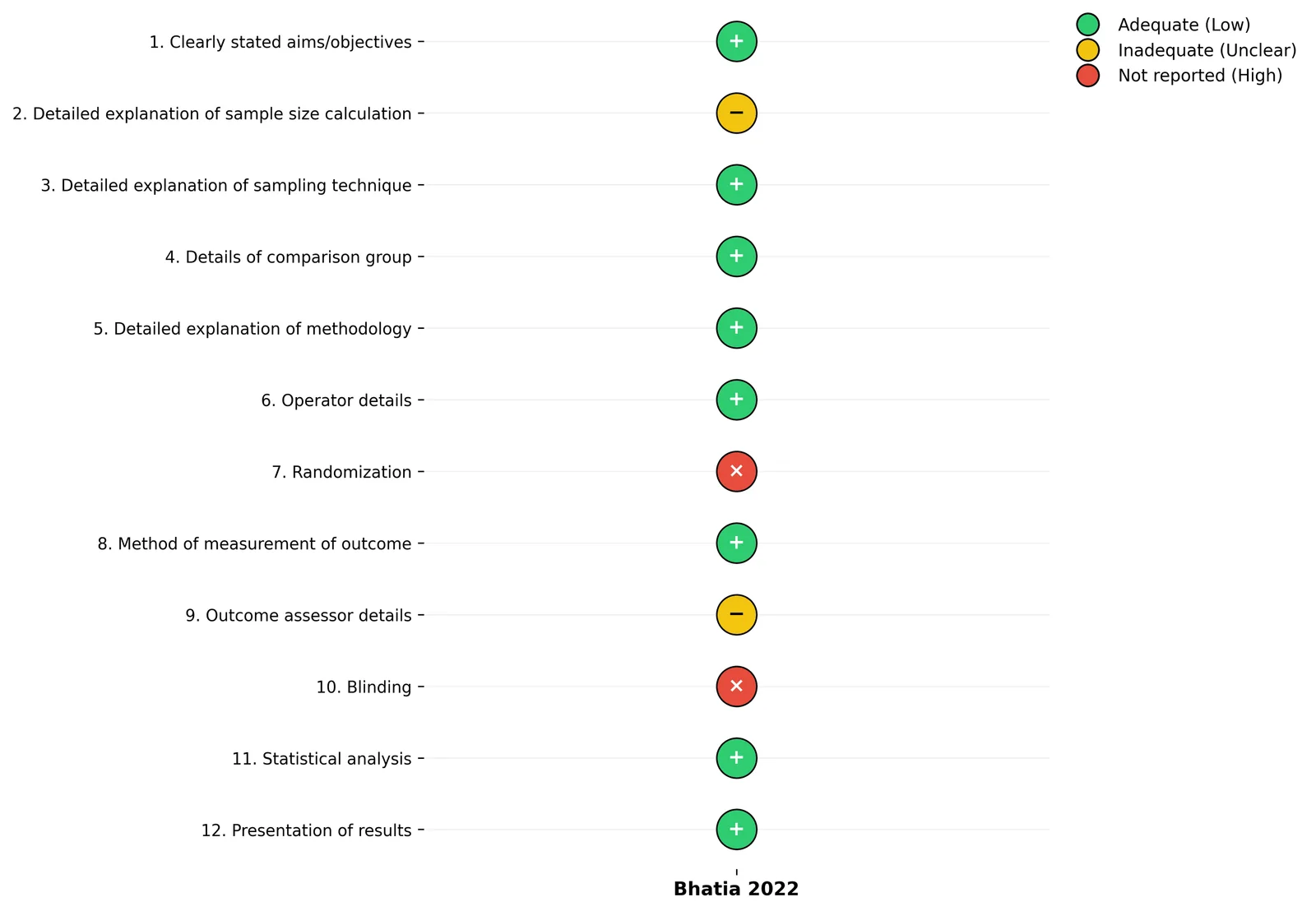
QUIN-tool appraisal for the included in vitro study demonstrating a final score of 75% (low risk of bias) [43]

The methodological quality of the 16 clinical trials was appraised using the Cochrane RoB 2. Overall, 14 trials (88%) were judged to be at low risk of bias, while 2 trials (12%) raised some concerns. Specifically, Olegário et al. were flagged for concerns regarding outcome measurement (Domain 4), and Sengul and Gurbuz [42] presented concerns regarding the randomization process (Domain 1). No trial was rated as having a high risk of bias in any domain (Figures 2-3). The single included in vitro study (Bhatia et al. [43]) was assessed using the QUIN tool. It achieved a final score of 75%, corresponding to a low risk of bias, despite inadequate/unclear reporting of the sample size calculation and outcome assessor blinding (Figure 4).

Primary Outcome: Clinical Success and Retention

**Figure 5:**
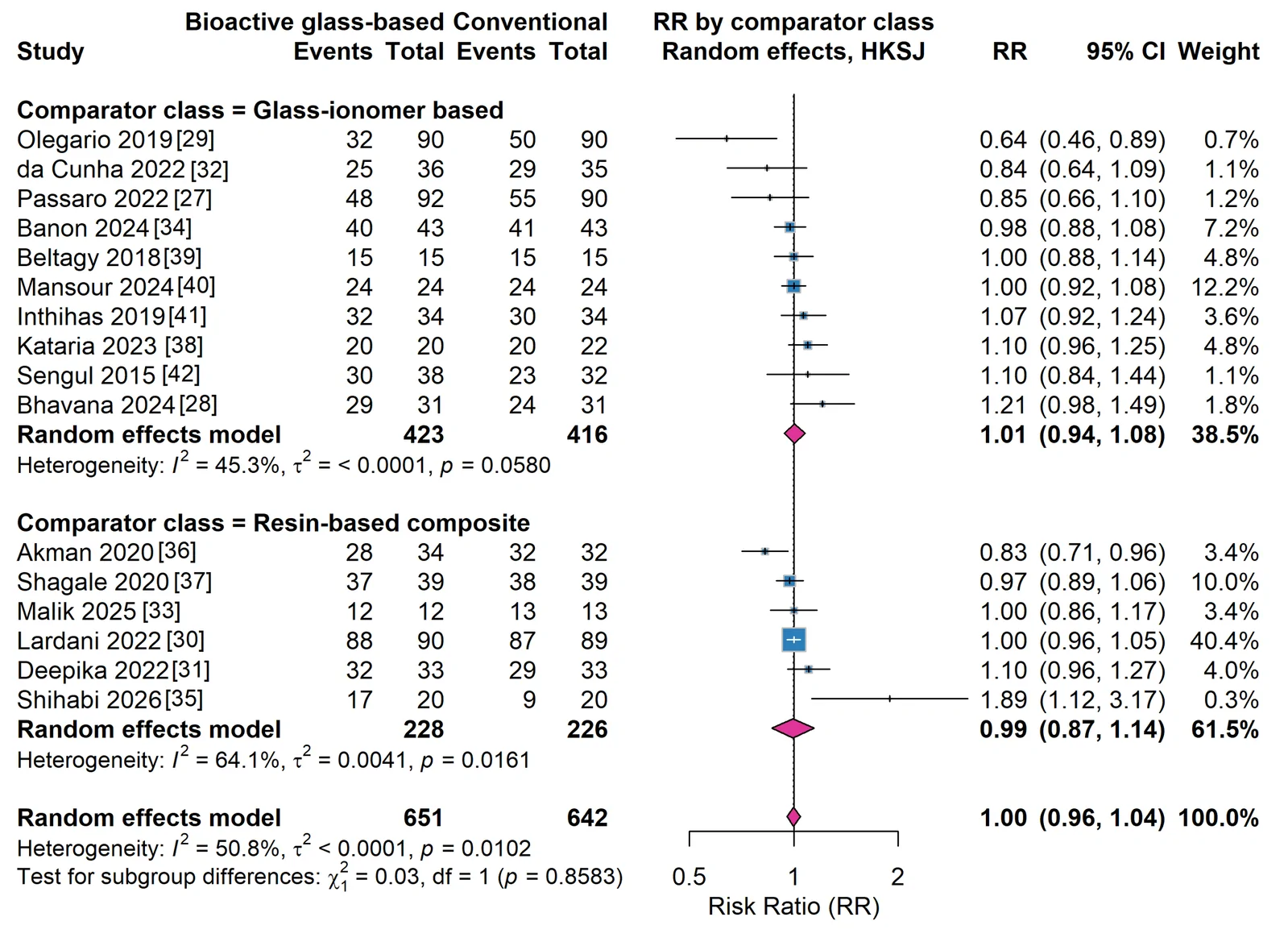
RE meta-analysis (REML, HKSJ adjustment) The forest plot compares clinical success/retention between bioactive and conventional materials, stratified by comparator class (glass-ionomer-based vs. resin-based composite) [27-42]. RR: risk ratio, HKSJ: Hartung-Knapp-Sidik-Jonkman, CI: confidence interval, REML: restricted maximum likelihood, RE: random-effects

Data from 16 RCTs, encompassing 1,293 randomized participants (651 in bioactive groups and 642 in conventional groups) and 1,028 successful events, were pooled to evaluate overall clinical success and retention. The Mantel-Haenszel RE meta-analysis utilizing the REML estimator with HKSJ adjustment revealed no statistically significant difference in clinical success between bioactive materials and conventional materials (RR = 1.00; 95% CI: 0.96-1.04; p = 0.9635) (Figure 5).

Moderate statistical heterogeneity was observed among the pooled trials (I² = 50.8%; τ² < 0.0001; Cochran’s Q = 30.51, p = 0.0102). However, the 95% prediction interval (0.97-1.03) remained narrow and closely bound to the line of no effect, suggesting that future trials are highly likely to demonstrate equivalent clinical success between the two material classes.

Subgroup and Meta-Regression Analyses

To explore the moderate heterogeneity observed in the primary outcome, a pre-specified subgroup analysis was conducted based on the comparator class (glass-ionomer-based (RMGIC/HVGIC/compomer) vs. resin-based composite). The test for subgroup differences revealed no significant effect modification (χ12​ = 0.03, p = 0.8583). Bioactive materials performed equivalently when compared with both glass-ionomer-based controls (RR = 1.01; 95% CI: 0.94 to 1.08; 10 trials; I² = 45.3%) and resin-based composite controls (RR = 0.99; 95% CI: 0.87 to 1.14; 6 trials; I² = 64.1%) (Figure 5).

**Figure 6:**
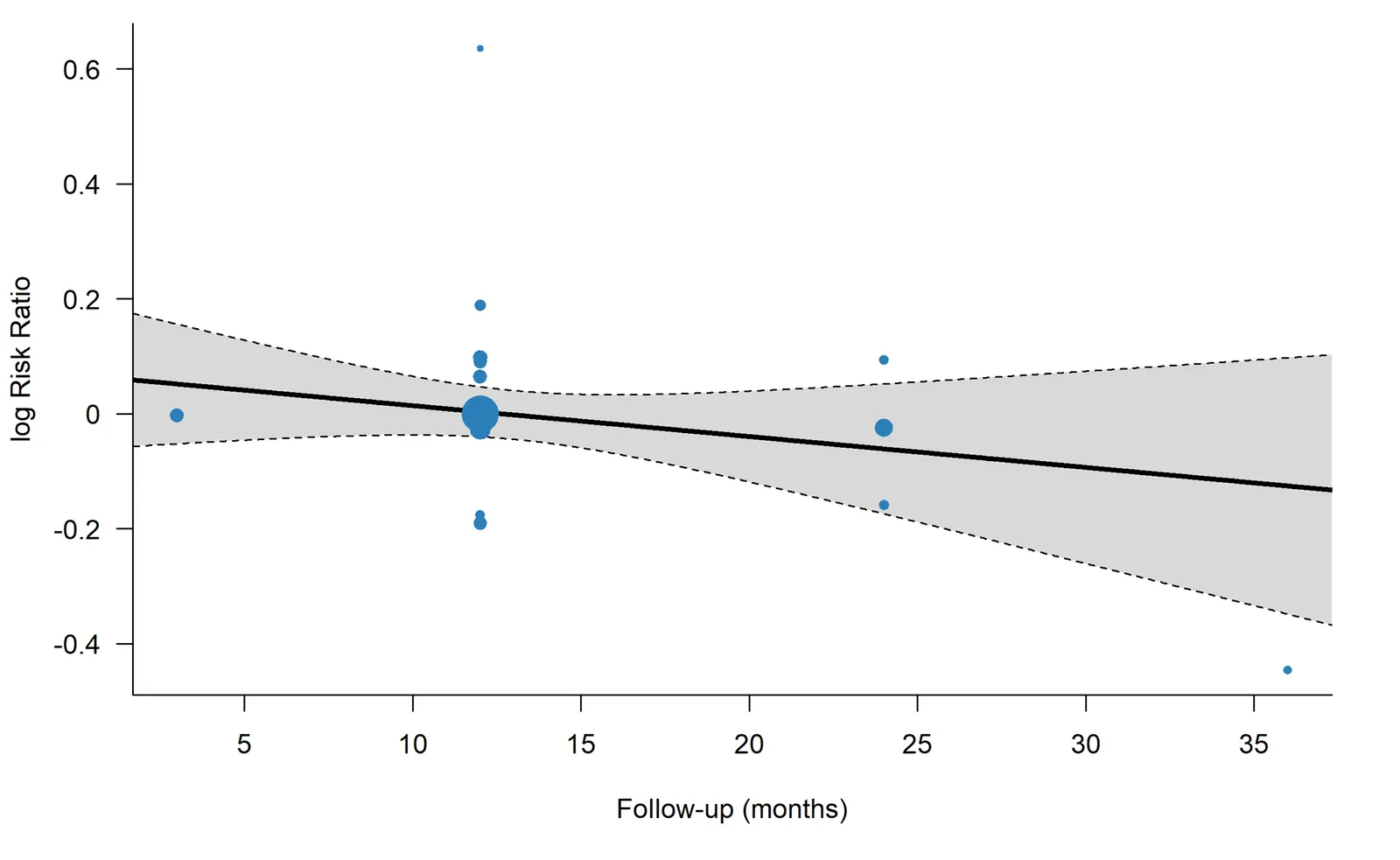
Meta-regression The bubble plot assesses the association between the log RR of clinical success and follow-up duration (months). RR: risk ratio

Furthermore, a RE meta-regression model was fitted on the log-RR scale to assess the impact of follow-up duration on the relative treatment effect. The follow-up duration (slope = -0.0054 per month; 95% CI: -0.015 to 0.004) did not significantly moderate the treatment effect (p = 0.2456), although it explained 78.4% of the residual heterogeneity (R2 = 78.4%) (Figure 6).

Sensitivity Analyses

**Figure 7:**
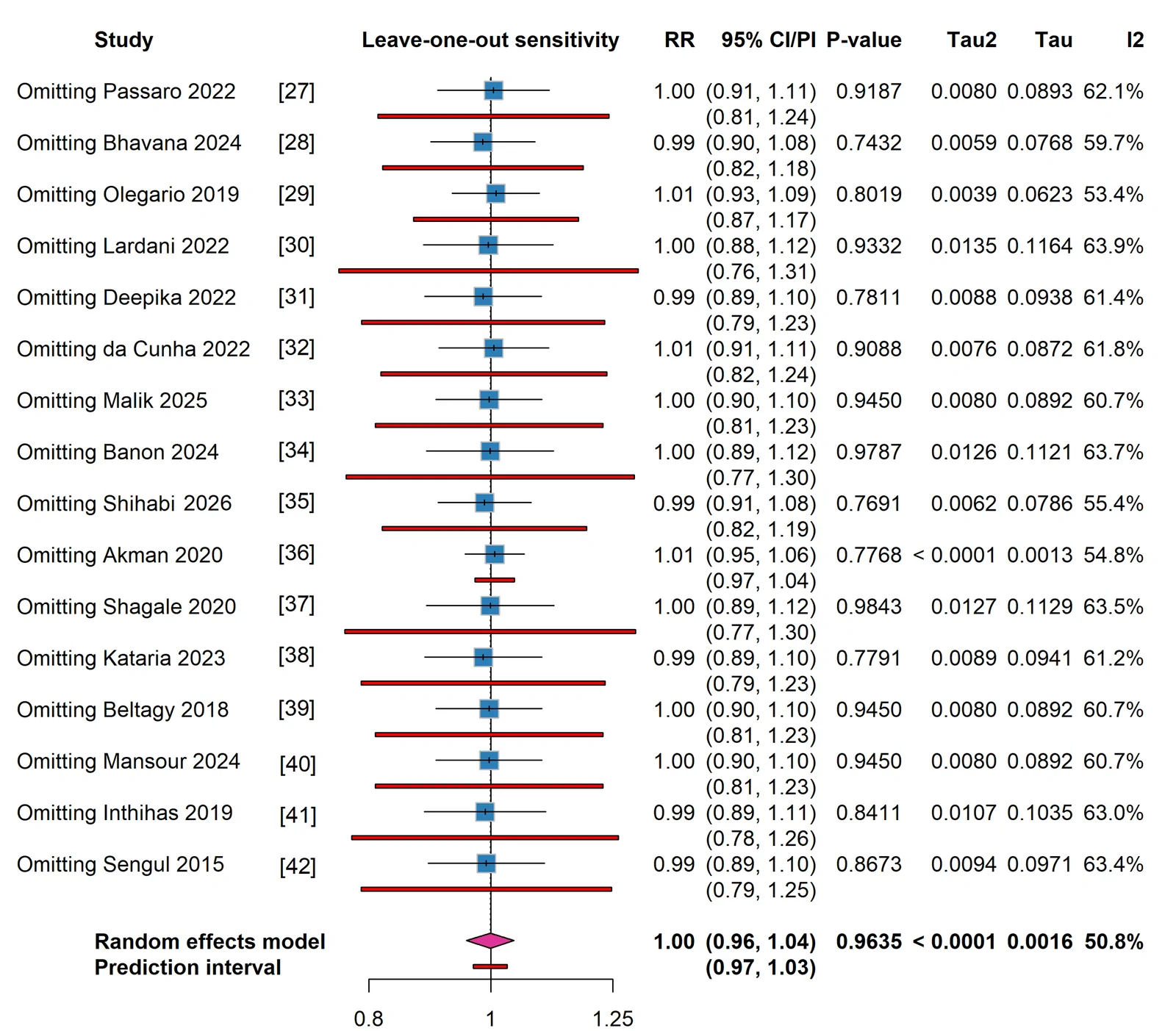
Leave-one-out sensitivity analysis The forest plot demonstrates the robustness of the pooled RR for clinical success [27-42]. RR: risk ratio

A leave-one-out sensitivity analysis confirmed the robustness of the primary pooled estimate, with the recalculated pooled RRs ranging between 0.99 and 1.01, and all corresponding CIs spanning the null value of 1.00. The omission of the study by Akman and Tosun [36] resulted in the most notable reduction in heterogeneity (I² decreased to 54.8%, τ² ≈ 0), identifying it as the primary contributor to the observed heterogeneity (Figure 7).

**Figure 8:**
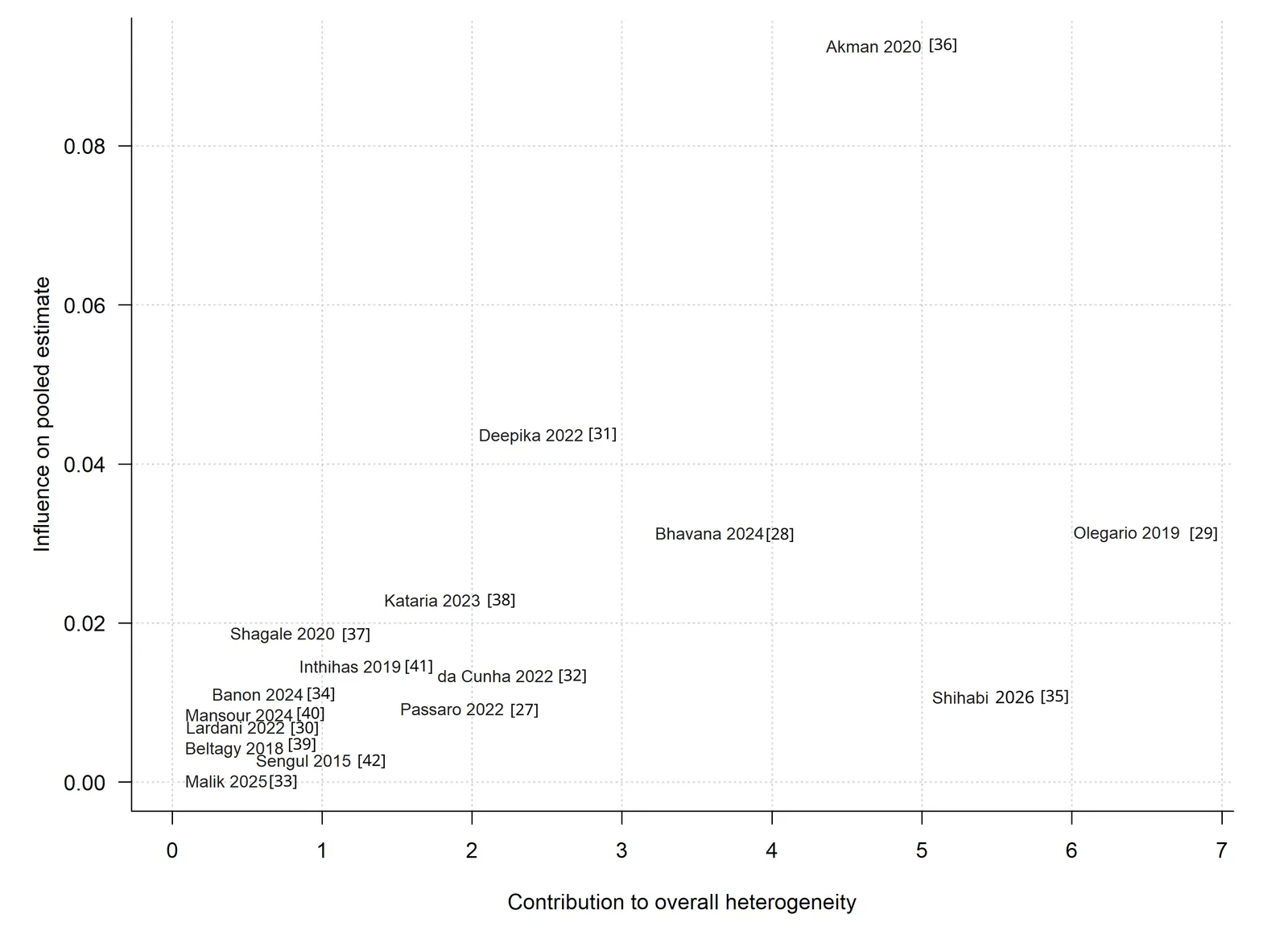
Baujat plot diagnosing the contribution of individual studies to overall heterogeneity vs. their influence on the pooled effect estimate [27-42]

**Figure 9:**
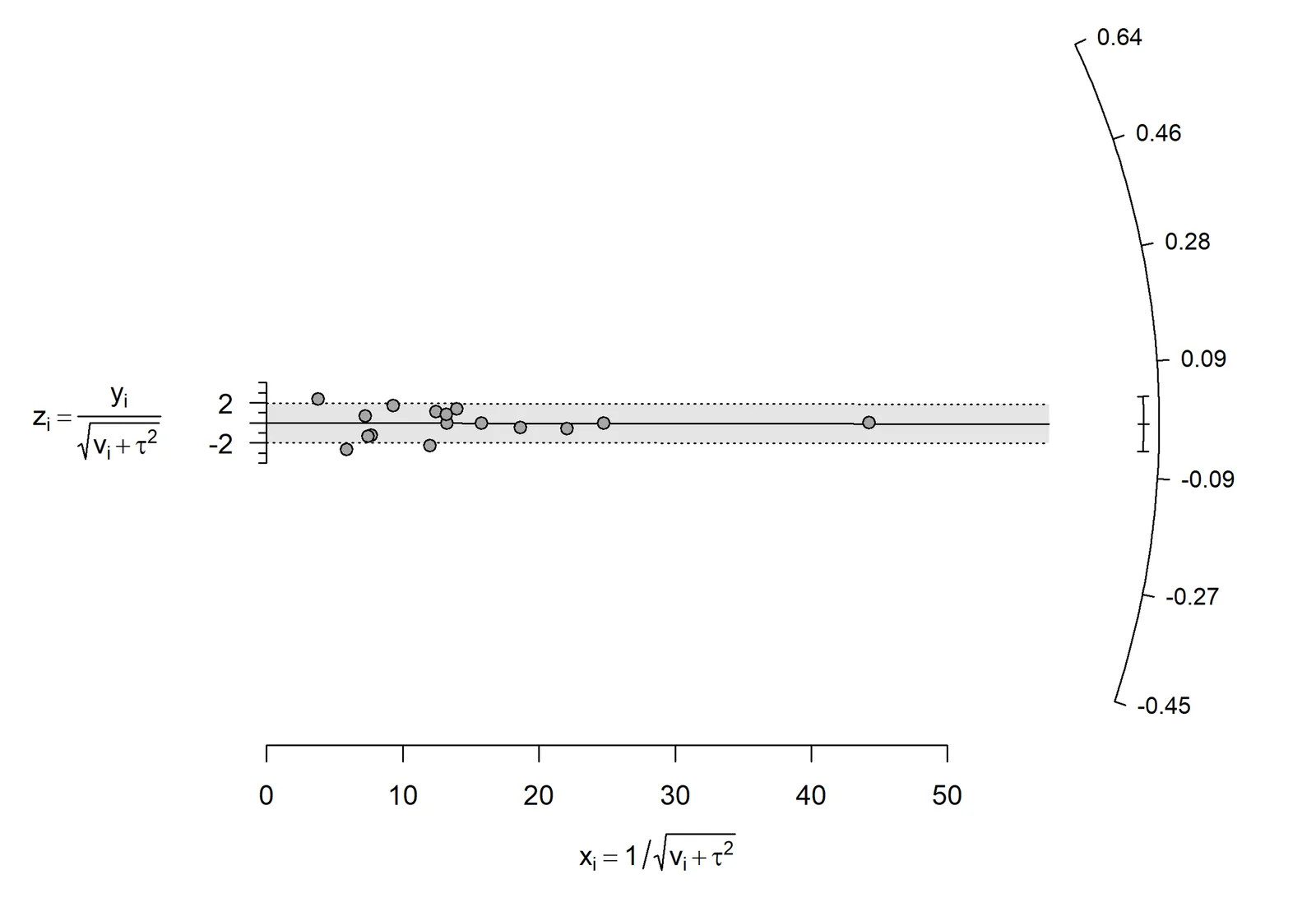
Radial (Galbraith) plot assessing heterogeneity and detecting potential outlier studies across the meta-analysis [27-42]

**Figure 10:**
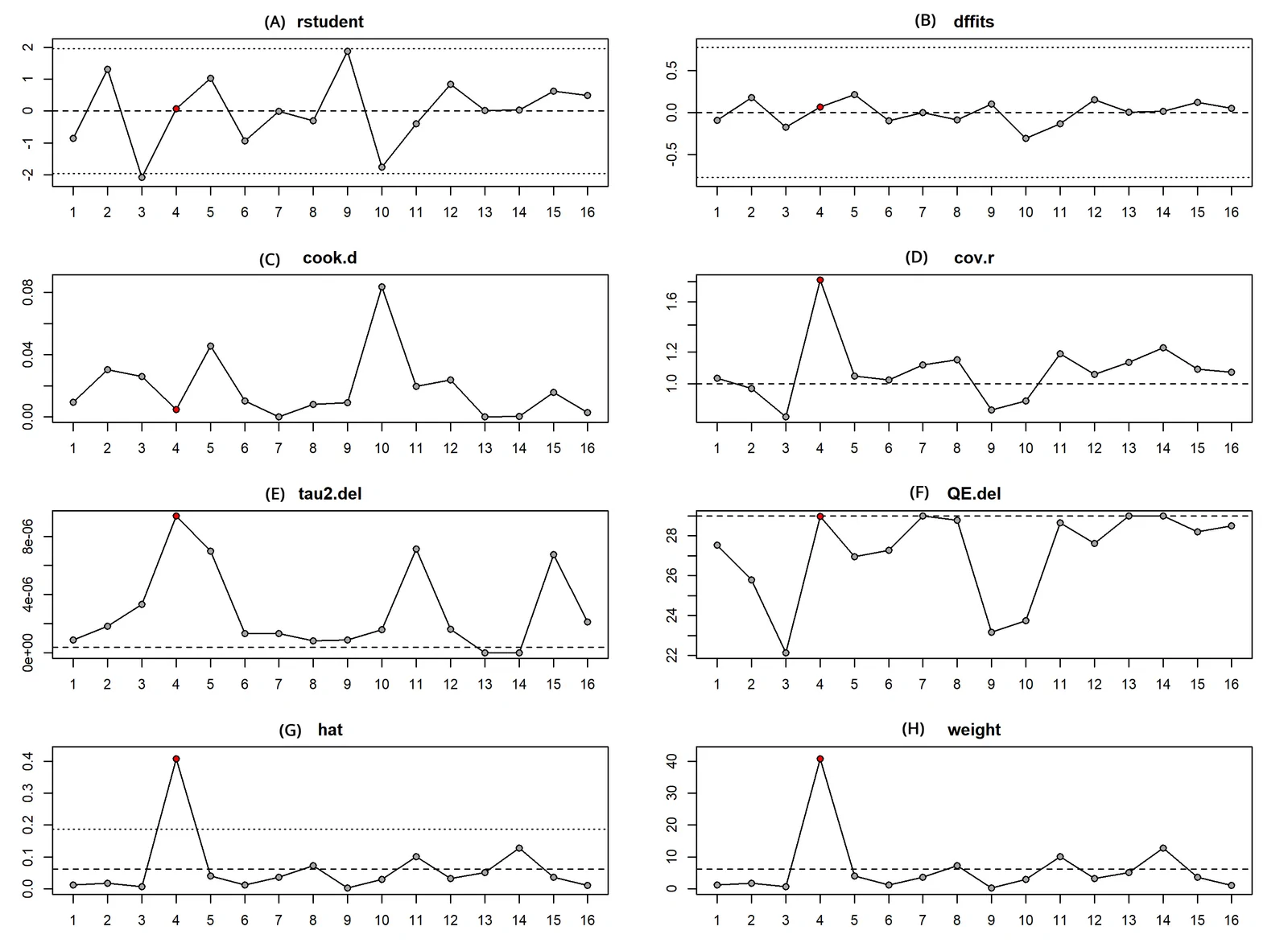
Comprehensive influence diagnostics for the primary meta-analysis model (A) Studentized residuals, (B) difference in fits (DFFITS), (C) Cook's distance, (D) covariance ratio, (E) τ² deletion, (F) Q deletion, (G) hat values, and (H) weights.

**Figure 11:**
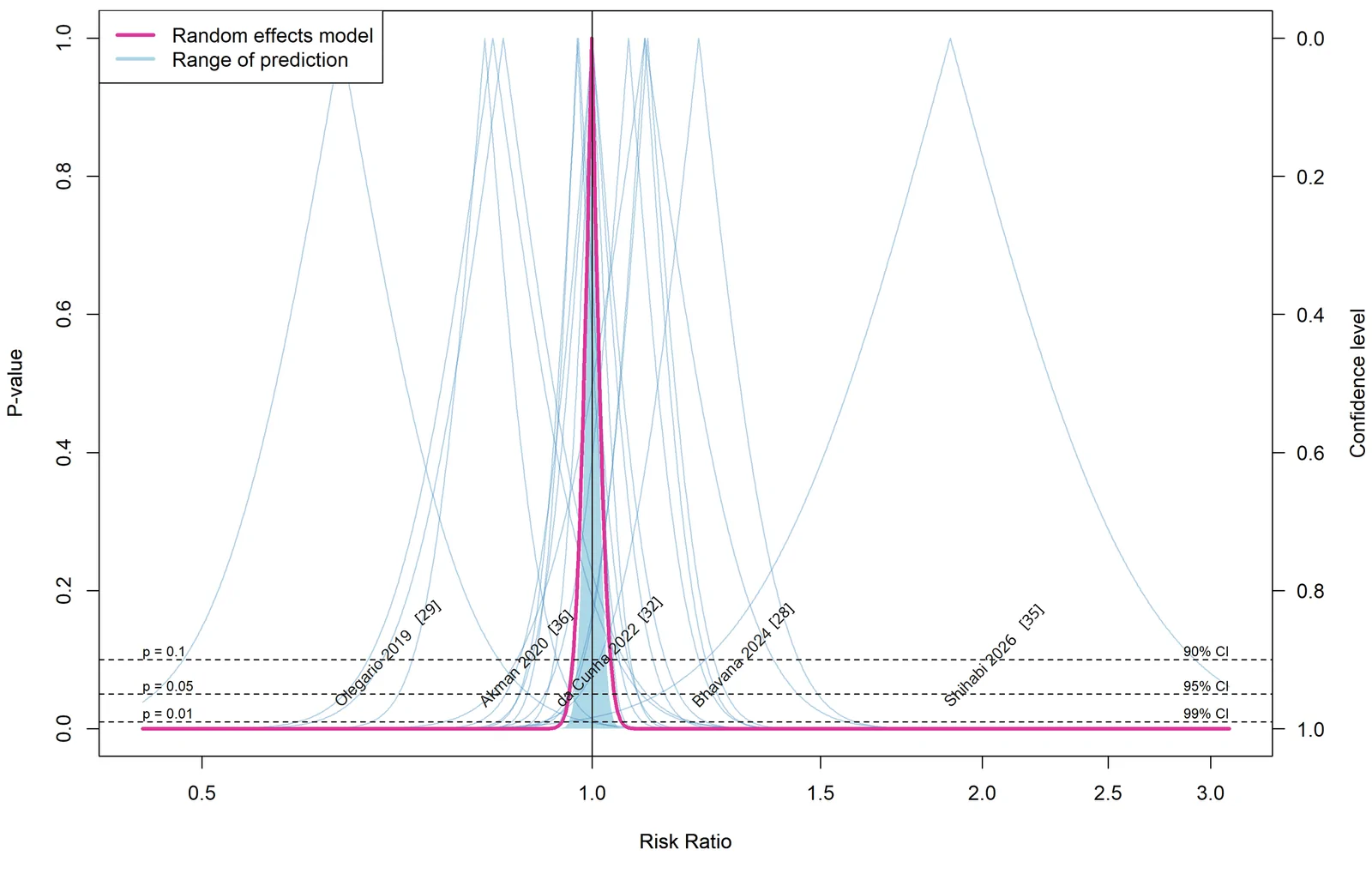
Drapery plot illustrating p-value functions and CIs across all candidate effect sizes CIs: confidence intervals

Further influence diagnostics were generated utilizing the metafor and dmetar packages in R, including Baujat plots (Figure 8), Galbraith radial plots (Figure 9), and standard diagnostic metrics (e.g., Cook's distance, difference in fits (DFFITS)) (Figure 10), which corroborated the stability of the model, confirming that no single study disproportionately skewed the overall conclusion. The drapery plot further illustrated the robust density of p-value functions centered around the null effect (Figure 11).

Publication Bias

**Figure 12:**
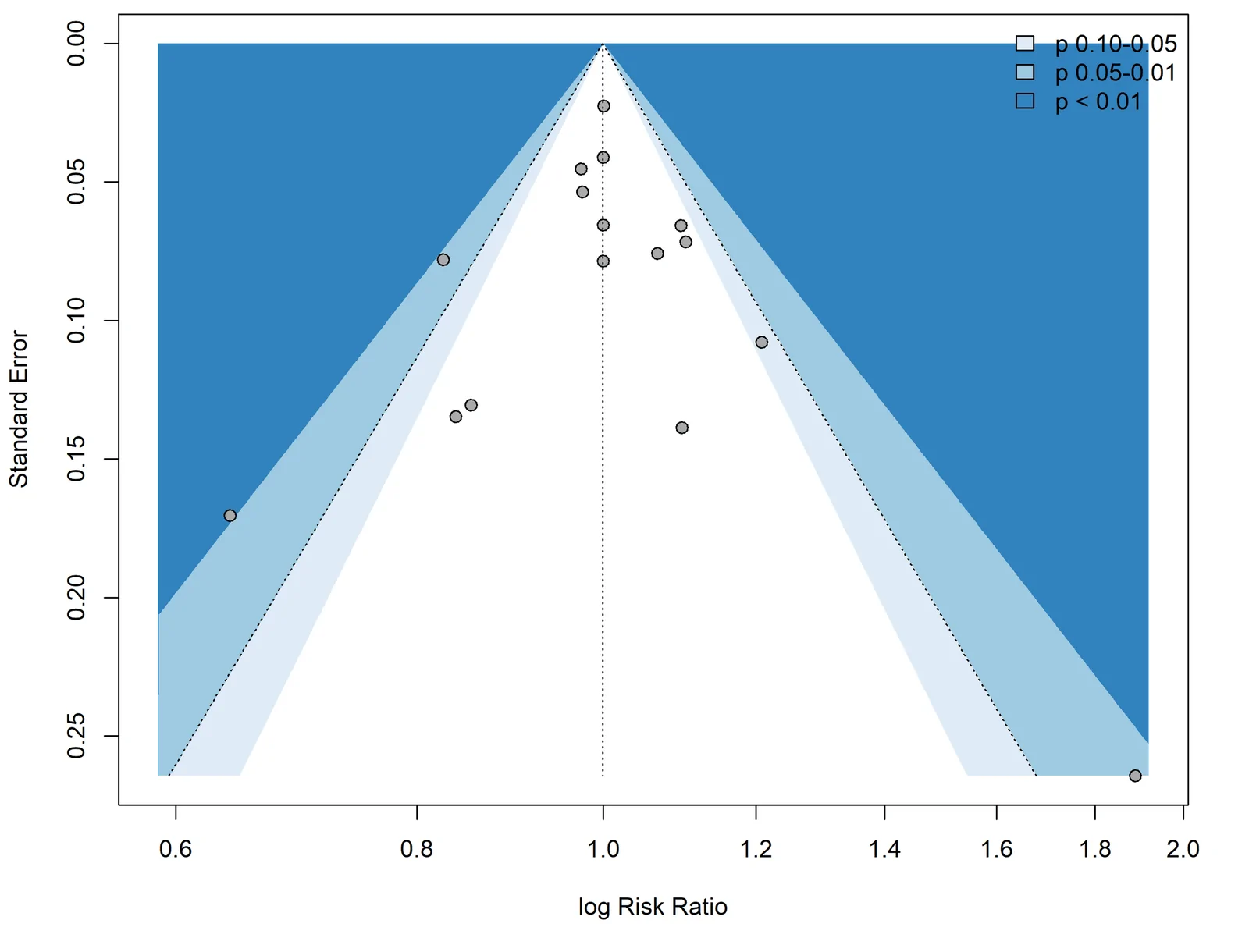
Contour-enhanced funnel plot assessing publication bias and small-study effects for the primary outcome of clinical success

Small-study effects and publication bias for the primary outcome (k = 16) were visually evaluated using a contour-enhanced funnel plot (Figure 12). The plot demonstrates a symmetric distribution of the study estimates within the regions of non-significance (white area). This visual symmetry was statistically confirmed by Egger’s linear regression test, which showed no evidence of funnel plot asymmetry (t = 0.02, df = 14, p = 0.9822; bias estimate = 0.02, SE = 0.679).

Secondary and In Vitro Outcomes

**Figure 13:**
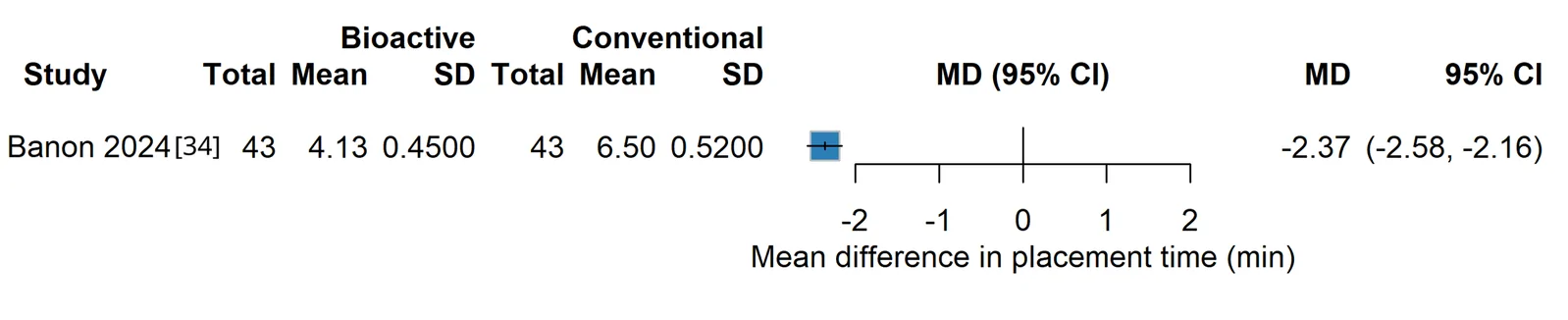
Forest plot depicting the MD in operator placement time (minutes) based on data from a single trial MD: mean difference, SD: standard deviation, CI: confidence interval [34]

Data about operator placement time were reported in a single trial (Banon et al. [34]). The use of the bioactive material resulted in a significantly faster operator placement time than the conventional compomer, with an MD of -2.37 minutes (95% CI: -2.58 to -2.16; p < 0.0001) (Figure 13).

**Figure 14:**
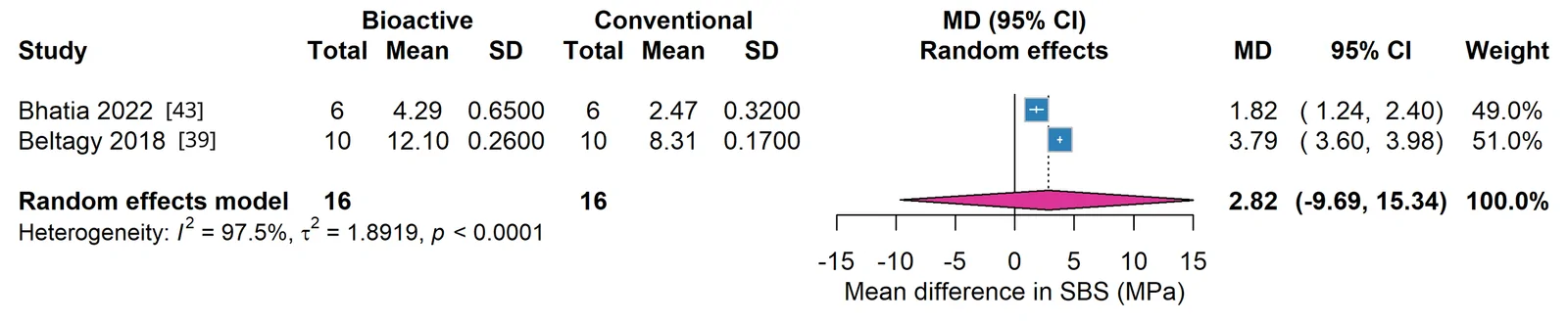
Forest plot of the RE meta-analysis comparing the in vitro SBS (MPa) between bioactive and conventional materials SBS: shear bond strength, MD: mean difference, SD: standard deviation, CI: confidence interval, RE: random-effects [39,43]

Regarding in vitro shear bond strength (SBS), data from two studies (Bhatia et al. [43] and Beltagy and Elhatery [39]; n = 16 specimens per group) were pooled. The RE meta-analysis favored bioactive materials (MD = 2.82 MPa); however, this result was highly imprecise and not statistically significant (95% CI: -9.69 to 15.34; p = 0.2136). Extreme statistical heterogeneity was observed for this surrogate outcome (I² = 97.5%; τ² = 1.89; p < 0.0001) (Figure 14).

Certainty of Evidence (GRADE)

The certainty of the evidence was appraised using the GRADE framework. For the primary outcome of overall clinical success/retention, the certainty of evidence was rated as high, driven by a large body of low-risk RCTs, precise null estimates, and no evidence of publication bias. The certainty of evidence for marginal integrity, secondary caries, and operator placement time was rated as low due to sparse data, reliance on single studies, and narrative syntheses. The evidence supporting the in vitro SBS was rated as very low due to extreme inconsistency and serious indirectness inherent to laboratory surrogate endpoints. A comprehensive GRADE summary of the findings is presented in Table 2.

**Table 2:** GRADE summary of findings Primary outcome: pooled RR 1.00 (95% CI 0.96 to 1.04), RE REML model with HKSJ adjustment; I² = 50.8%; 95% prediction interval 0.97 to 1.03; Egger p = 0.9822 (no small-study effects). The assumed control (conventional) success risk was 81 per 100. Risk of bias: 14/16 trials had a low risk, two had some concerns (RoB 2), and the in vitro study had a low risk (QUIN 75%). Secondary and in vitro outcomes are based on few studies and are interpreted as exploratory; the in vitro SBS estimate is imprecise (95% CI crosses 0) despite a consistent direction favoring bioactive materials. CI: confidence interval, RR: risk ratio, MD: mean difference, PI: prediction interval, RCT: randomized controlled trial, I²: inconsistency statistic, GRADE: Grading of Recommendations Assessment, Development, and Evaluation, REML: restricted maximum likelihood, HKSJ: Hartung-Knapp-Sidik-Jonkman, RE: random-effects, SBS: shear bond strength

Outcome (follow-up)	№ participants (studies)	Relative effect (95% CI)	Anticipated absolute effects	Direction	Certainty (GRADE)	Reason(s) for downgrading
Overall clinical success/retention (3-36 mo)	1293 (16 RCTs)	RR 1.00 (0.96 to 1.04)	81 per 100 with conventional; −3 per 1000 (−41 to +38)	No difference	⊕⊕⊕⊕ HIGH	No serious limitations (I^2 ^= 50.8%, PI narrow)
Marginal integrity	- (multiple RCTs)	Narratively favors bioactive (not pooled)	-	Favours bioactive	⊕⊕○○ LOW	Inconsistency + imprecision (sparse pooling)
Secondary caries (incidence)	- (subset of RCTs)	No significant difference reported	-	No difference	⊕⊕○○ LOW	Indirectness + imprecision
Operator placement time (min)	86 (1 RCT, Banon 2024)	MD −2.37 min (−2.58 to −2.16)	2.37 min faster placement	Favours bioactive	⊕⊕○○ LOW	Very serious imprecision (single small study)
In vitro SBS (MPa)	32 specimens (2 in vitro)	MD 2.82 MPa (−9.69 to 15.34)	Higher bond strength in vitro	Favours bioactive	⊕○○○ VERY LOW	Very serious indirectness (in vitro surrogate); I^2 ^= 97.5%

Discussion

This systematic review and meta-analysis synthesized the highest-quality available evidence to determine the clinical efficacy of bioactive materials vs. conventional materials in primary dentition. Across 16 RCTs encompassing 1,293 pediatric participants, the findings demonstrated clinical equipoise: bioactive materials performed equivalently to conventional materials in terms of overall clinical success and restoration retention (RR = 1.00; 95% CI: 0.96 to 1.04). This primary outcome was statistically robust, corroborated by extensive sensitivity and influence diagnostics, and achieved a high certainty rating under the GRADE framework. Furthermore, secondary analyses highlighted the potential operational advantages of bioactive materials, specifically a significant reduction in clinical operator placement time.

The equivalent clinical success observed in this meta-analysis bridges the gap between the theoretical advantages of bioactive materials and their observed clinical performance. Primary dentition presents a hostile biomechanical and chemical environment for restorations, characterized by thin enamel, less retentive cavity forms, and high caries susceptibility [44]. Conventional resin composites, while aesthetically superior and mechanically strong, lack intrinsic anti-cariogenic properties and are sensitive to moisture contamination, a frequent occurrence in pediatric patients with limited compliance [45]. Conventional GICs offer moisture tolerance and fluoride release but suffer from inferior fracture toughness and wear resistance in load-bearing Class II cavities [46].

Bioactive materials (e.g., ACTIVA BioACTIVE, Cention N, and Equia Forte) were engineered to combine the strength of resin matrices with the dynamic ion-exchanging capabilities of glass ionomers [30,31]. The pooled data suggest that these materials can achieve this hybridization. When stratified by comparator class, bioactive materials performed equally well against both glass-ionomer-based controls (RR = 1.01) and resin-based composites (RR = 0.99), indicating that the inclusion of bioactive glass fillers and unique resin matrices (e.g., shock-absorbing rubberized resins or alkasites) provides sufficient mechanical integrity to match composites, while providing the marginal seal and ionic exchange necessary to match or marginally exceed RMGICs [27,38].

Of critical importance to pediatric clinicians is the secondary finding regarding operator placement time. Data from Banon et al. [34] demonstrated a 2.37-minute reduction in operator placement time when using a bulk-fill bioactive composite compared to a conventionally layered compomer. Minimizing chair time is paramount in pediatric behavioral management. A reduction of nearly two minutes during the most technique-sensitive phase of the appointment minimizes the risk of salivary contamination, reduces child fatigue, and indirectly contributes to the high clinical success rates observed. While in vitro data (e.g., SBS) favored bioactive materials (MD = 2.82 MPa), the extreme heterogeneity (I² = 97.5%) and serious indirectness of these laboratory surrogates highlight the need to rely on robust in vivo clinical endpoints for decision-making [43,47].

Strengths and Limitations

The principal strength of this meta-analysis was its rigorous methodological execution. By adhering to the PRISMA 2020 guidelines and restricting inclusion to RCTs (for clinical outcomes), we minimized confounding variables and selection bias. The employment of the REML estimator with the HKSJ adjustment provided a highly conservative and reliable CI, protecting against the false-positive inflation often seen in standard DerSimonian-Laird RE models [22]. Additionally, the lack of publication bias, as confirmed by contour-enhanced funnel plots and Egger’s test, bolstered the validity of the synthesized effect size.

However, this study has certain limitations that must be acknowledged. First, moderate statistical heterogeneity (I² = 50.8%) was observed for the primary outcome. While meta-regression identified follow-up duration as a partial driver of this variance (nominally accounting for 78.4% of residual heterogeneity), differences in cavity classifications (Class I vs. Class II) and variations in the specific proprietary formulations of bioactive materials contributed to this inconsistency. Second, the evaluation of secondary clinical outcomes (e.g., marginal discoloration and incidence of secondary caries) was hindered by inconsistent reporting metrics across the primary trials, preventing formal meta-analytic pooling. Finally, most of the included RCTs had follow-up periods ranging from 12 to 24 months. While adequate for early performance assessment, primary molars often require restorations to survive for three to five years prior to natural exfoliation [44].

Future Directions

Future RCTs should standardize outcome reporting by adopting core outcome sets for pediatric restorative dentistry. Studies with extended follow-up durations (36-60 months or until natural tooth exfoliation) are required to ascertain whether the remineralizing properties of bioactive materials confer a long-term advantage in preventing secondary caries compared to inert resins. Furthermore, high-quality health economic evaluations (cost-effectiveness analyses) are needed to determine whether the workflow efficiencies (i.e., reduced operator placement time) and clinical equivalency of bioactive materials justify their implementation in public health and high-volume pediatric settings.

## Conclusions

Bioactive glass-based materials are a clinically sound, non-inferior alternative to conventional materials for managing carious primary teeth. By providing equivalent overall clinical success and restoration retention, alongside potential advantages in operator efficiency (e.g., significantly reduced operator placement time) and moisture tolerance, bioactive materials represent a highly efficacious and biomimetic addition to the pediatric dental armamentarium. However, while short- to medium-term results are promising, future long-term RCTs utilizing standardized core outcome sets are needed to fully substantiate their longitudinal anti-cariogenic benefits and cost-effectiveness in diverse clinical settings.
